# PBPK Modeling and Simulation of Antibiotics Amikacin, Gentamicin, Tobramycin, and Vancomycin Used in Hospital Practice

**DOI:** 10.3390/life11111130

**Published:** 2021-10-23

**Authors:** Abigail Ferreira, Helena Martins, José Carlos Oliveira, Rui Lapa, Nuno Vale

**Affiliations:** 1 OncoPharma Research Group, Center for Health Technology and Services Research (CINTESIS), Rua Doutor Plácido da Costa, 4200-450 Porto, Portugal; abigail.ferreira@fc.up.pt; 2 LAQV/REQUIMTE, Laboratory of Applied Chemistry, Department of Chemical Sciences, Faculty of Pharmacy, University of Porto, Rua de Jorge Viterbo Ferreira, 228, 4050-313 Porto, Portugal; laparuas@ff.up.pt; 3 Departament of Pathology, Clinical Chemistry Service, Centro Hospitalar Universitário do Porto (CHUP), 4099-001 Porto, Portugal; helena.martins.sqc@chporto.min-saude.pt (H.M.); director.sqc@chporto.min-saude.pt (J.C.O.); 4 Department of Community Medicine, Health Information and Decision (MEDCIDS), Faculty of Medicine, University of Porto, 4200-319 Porto, Portugal

**Keywords:** PBPK modeling, therapeutic drug monitoring, antibiotics use, amikacin, gentamicin, tobramycin, vancomycin, GastroPlus™

## Abstract

The importance of closely observing patients receiving antibiotic therapy, performing therapeutic drug monitoring (TDM), and regularly adjusting dosing regimens has been extensively demonstrated. Additionally, antibiotic resistance is a contemporary concerningly dangerous issue. Optimizing the use of antibiotics is crucial to ensure treatment efficacy and prevent toxicity caused by overdosing, as well as to combat the prevalence and wide spread of resistant strains. Some antibiotics have been selected and reserved for the treatment of severe infections, including amikacin, gentamicin, tobramycin, and vancomycin. Critically ill patients often require long treatments, hospitalization, and require particular attention regarding TDM and dosing adjustments. As these antibiotics are eliminated by the kidneys, critical deterioration of renal function and toxic effects must be prevented. In this work, clinical data from a Portuguese cohort of 82 inpatients was analyzed and physiologically based pharmacokinetic (PBPK) modeling and simulation was used to study the influence of different therapeutic regimens and parameters as biological sex, body weight, and renal function on the biodistribution and pharmacokinetic (PK) profile of these four antibiotics. Renal function demonstrated the greatest impact on plasma concentration of these antibiotics, and vancomycin had the most considerable accumulation in plasma over time, particularly in patients with impaired renal function. Thus, through a PBPK study, it is possible to understand which pharmacokinetic parameters will have the greatest variation in a given population receiving antibiotic administrations in hospital context.

## 1. Introduction

Therapeutic drug monitoring (TDM) was implemented in the 1960s to improve patient care and clinical outcome, and specializes in the measurement of circulating drug concentrations to adjust dosing regimens, so as to reach a defined target exposure associated with optimal efficacy and minimal toxicity [1,2]. Since then, some pharmacological properties have been identified (particularly narrow therapeutic window and significant interpatient variability) and the cases that required such dosage individualization have been comprehensively reviewed. TDM is now indicated and recommended for critically ill patients undergoing sufficiently long treatment to justify dosage adjustment [3], whether with anticancer drugs [4], anti-infectives [5], antiretrovirals [6], biologic therapeutic agents [7] or psychotropic agents [8], etc.

Importantly for this work, therapeutic drug monitoring and dosage adjustments are crucial for antibiotics, and have confirmed beneficial results [9,10,11,12,13,14,15]. The four antibiotics studied here, amikacin, gentamicin, tobramycin, and vancomycin, are the most frequently monitored in inpatients, which can be explained by their narrow therapeutic indexes and potential to cause adverse effects, namely nephrotoxicity, particularly in prolonged treatments [15,16,17]. The value of TDM of these antibiotics has been extensively demonstrated [13,14,18,19,20,21,22,23,24,25,26,27].

Furthermore, the increasing resistance rates to antimicrobial agents are a pressing problem that has been indefectibly associated with the inappropriate administration and overuse of these drugs, which has accelerated this process over the past 80 years. About half of the antimicrobial agents prescribed to hospital inpatients are considered inappropriate [28]. Different approaches have been promoted, and antimicrobial stewardship is currently considered the most promising and has been promoted for all hospitals and health care facilities [29,30,31,32]. In 2014, the U.S. Centers for Disease Control and Prevention (CDC) identified seven core elements of antibiotic stewardship and recommend that all hospitals have an antibiotic stewardship program (ASP). Tracking (monitoring process measures), reporting information on antibiotic use and resistance, and education of clinicians and health care providers are three of these core elements [33].

These four antimicrobial agents are an example of antibiotics that have been reserved for the treatment of severe infections, in an attempt to save these valuable drugs from the threat of selection of resistant bacteria. Healing these infections often requires long periods of time of antibiotic therapy and hospitalization. This poses serious concerns, namely regarding plasma accumulation of these drugs, toxicity, and impairment of renal function. Kidneys’ deterioration can be an effect of the disease or a consequence of long-term treatment. Since these antibiotics are mainly renally eliminated and their clearance is an important factor to consider when determining treatment regimens, therapeutic drug monitoring and dose adjustment are imperative to ensure a good clinical outcome of these patients.

In this work, clinical data from a cohort of Portuguese inpatients with severe infections were collected and analyzed, and the influence of therapeutic regimens, biological sex, total body weight, and renal function on the pharmacokinetic (PK) profile was studied for four antibiotics: aminoglycosides amikacin, gentamicin, and tobramycin, and glycopeptide vancomycin. PK studies including physiologically based pharmacokinetic (PBPK) modeling and simulations were performed using GastroPlus™, based on our experience and previous works that have highlighted the value of in silico tools [34,35,36].

## 2. Materials and Methods

### 2.1. Study Population

Demographic data (biological sex, age, weight, and height) and clinical information were collected from 82 inpatients hospitalized in *Centro Hospitalar Universitário do Porto* (CHUP, Portugal) with severe systemic infections of different etiologies. These patients received intravenous (IV) treatment with one of four antibiotics: aminoglycoside amikacin, gentamicin, or tobramycin, or glycopeptide vancomycin. Drugs were administered via 30-min infusions in the case of aminoglycosides and 1-h infusions for vancomycin. Throughout the hospitalization and treatment period, trough and peak antibiotic concentrations as well as serum creatinine levels were determined to adjust posology and evaluate renal function (estimating creatinine clearance using the Cockcroft–Gault formula [37]). Recommended and followed treatment regimens were also recorded. The gathered information is presented in Supplementary Table S1 and summarized in Table 1.

### 2.2. Pharmacokinetic Analysis Software

PBPK modeling and simulation software GastroPlus™ version 9.5 (Simulations Plus Inc., Lancaster, CA, USA) was used for the prediction of PK parameters and generation of simulated human plasma concentration profiles. Some physicochemical properties of the four antibiotics and input parameters used in the simulations are presented in Table 2.

GastroPlus™, a powerful mechanistically based simulation and modeling software, has been specifically developed for pharmaceutical research. Physiological parameters of several species, including human, are preinstalled, allowing for the amount of drug that is released, dissolved, and absorbed to be modeled for nine compartments, corresponding to different segments of the digestive tract, based on a set of differential equations. The Advanced Compartmental Absorption and Transit (ACAT) and Physiologically Based Pharmacokinetic (PBPK) models support model-based drug development throughout multiple stages of drug discovery, translational research, and clinical development, making it a powerful tool. As such, this software has been used in numerous research studies, but also by distinguished pharmaceutical companies and by the U.S. Food and Drug Administration, the U.S. Centers for Disease Control and Prevention, the U.S. National Institutes of Health, the U.S. National Cancer Institute, and the China Food and Drug Administration [45,46]. A PBPK analysis uses models and simulations that combine physiology, population, and drug characteristics to mechanistically describe the PK behavior of a drug. Throughout a drug’s life cycle, PBPK model predictions can be used to support decisions on whether, when, and how to conduct certain clinical pharmacology studies and to support dosing recommendations in product labeling. The predictive performance of PBPK models in specific clinical settings with heterogeneous and chronically ill patients characterized by numerous unknown individual and clinical factors can be developed [47,48,49,50,51].

### 2.3. PBPK Modeling and Simulation

Every simulation was performed to study the PK for 72 h after IV administrations of each antibiotic, over 30-min infusions for aminoglycosides amikacin, gentamicin and tobramycin, and 1-h infusions for vancomycin.

For each antibiotic, a customized PBPK model was built to represent the average of that subpopulation (receiving treatment with a particular antibiotic). The input parameters were age, total body weight, and renal clearance (Table 1). Different therapeutic regimens (doses and dosing intervals) were studied for each antibiotic, based on the most common schemes administered to the patients in CHUP. Then, the most recurrent therapeutic regimen was chosen for each antibiotic and the influence of biological sex, body weight and renal function was assessed. Customized PBPK models were set up for male and female individuals with the average characteristics of the subpopulation. The effect of body weight and renal function was studied, defining these parameters as the lower and upper limit of the observed range (Table 1), creating 4 additional tailored PBPK models to represent these different conditions: lowest and highest body weight, and lowest and highest renal function, estimated by creatinine clearance.

The available data only included measured concentrations from 2 time points: right before a dose and 1 h (aminoglycosides) or 3 h (vancomycin) after the beginning of an infusion. Since in GastroPlus™ the observed data can only be used as input referring to a single administration, this prevented a further refined parameterization and validation of the developed models. Nevertheless, the predicted pharmacokinetic parameters were reasonably consistent with the observed values after administration of all analyzed doses for these 4 antibiotics.

## 3. Results

### 3.1. Influence of Dose Regimens and Biological Sex

**Figure 1:**
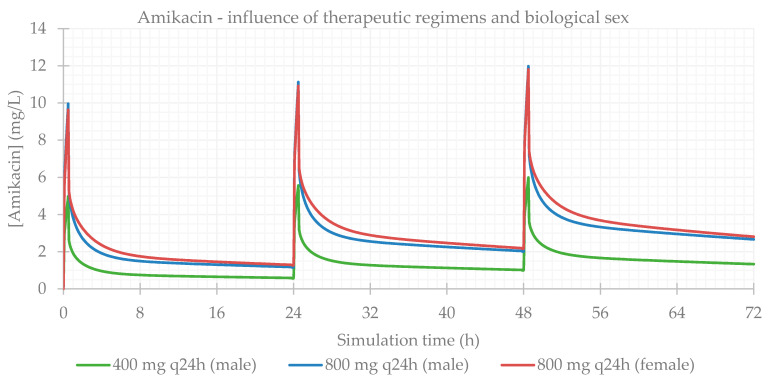
Amikacin plasma concentration–time profile showing the influence of therapeutic regimens and biological sex (considering the average of the study population: 57 years old, 66.0 kg, 169 cm, CL = 6.07 L/h).

**Figure 2:**
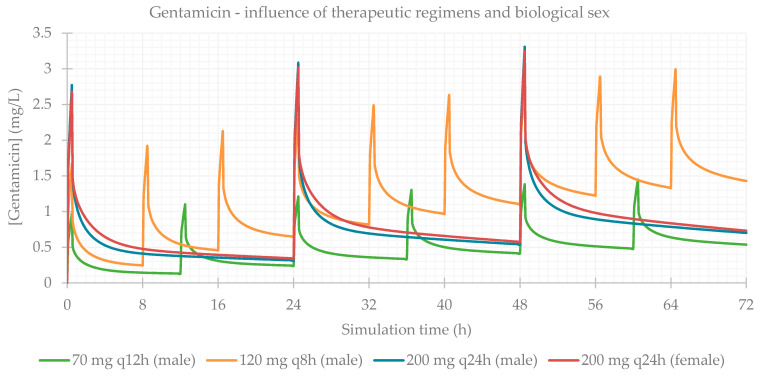
Gentamicin plasma concentration–time profile showing the influence of therapeutic regimens and biological sex (considering the average of the study population: 58 years old, 66.0 kg, 165 cm, CL = 5.94 L/h).

**Figure 3:**
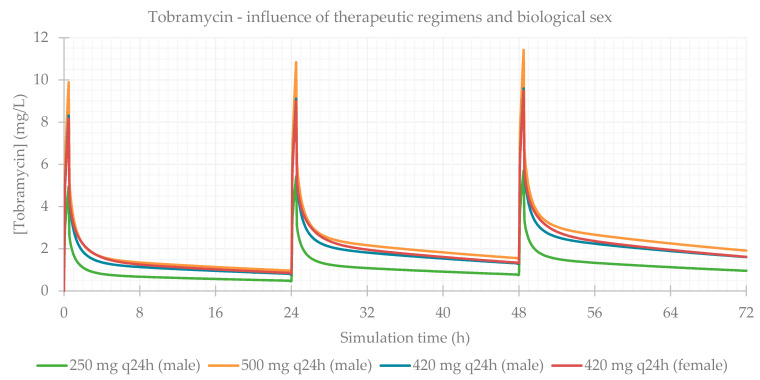
Tobramycin plasma concentration–time profile showing the influence of therapeutic regimens and biological sex (considering the average of the study population: 15 years old, 33.0 kg, 146 cm, CL = 6.01 L/h).

**Figure 4:**
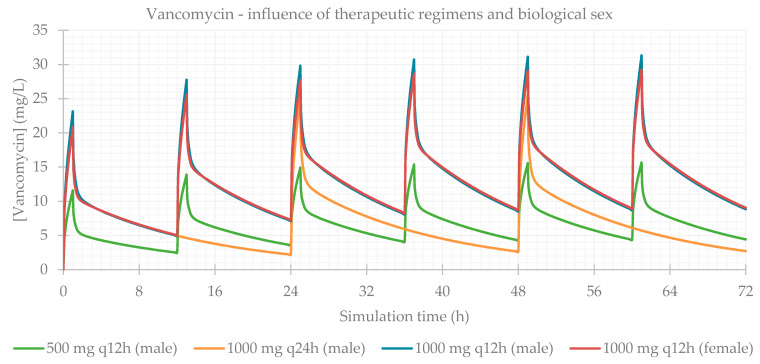
Vancomycin plasma concentration–time profile showing the influence of therapeutic regimens and biological sex (considering the average of the study population: 63 years old, 69.0 kg, 163 cm, CL = 5.79 L/h).

Different therapeutic regimens (doses and dosing intervals) were studied for each antibiotic, based on the most common schemes administered to the patients in the study population. The influence of biological sex was also assessed. The simulation-calculated PK parameters are presented in Table 3 and the plasma concentration–time profiles showing the influence of these two factors are presented for each antibiotic in Figure 1, Figure 2, Figure 3 and Figure 4. As expected for IV infusions, the predicted fraction absorbed (Fa) and bioavailability (F) of all antibiotics were >99%, and a peak in antibiotic concentration was always predicted to be reached at the end of infusions. GastroPlus™ also calculated a direct proportionality of dose with C_max_ and AUC: doubling the dose increased these PK parameters by 2-fold. Additionally, with shorter time intervals between doses, antibiotic accumulation in plasma was more evident. Regarding biological sex, slight differences were observed between the profiles simulated for male and female subjects; a higher C_max_, but lower concentrations until following administration is predicted for male individuals, except for vancomycin, where only differences in C_max_ were noted.

### 3.2. Influence of Total Body Weight

**Figure 5:**
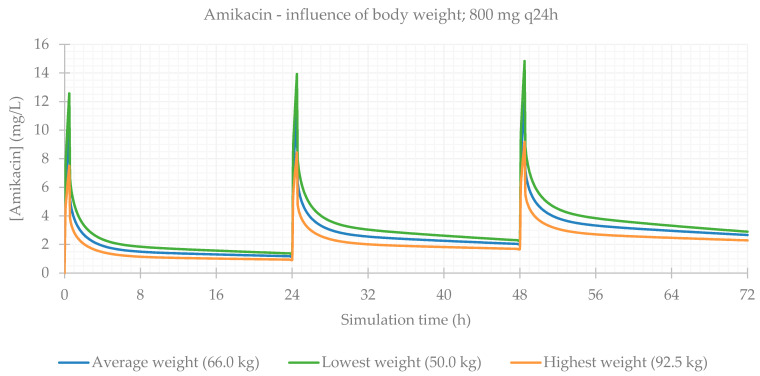
Amikacin plasma concentration–time profile showing the influence of body weight (for an 800 mg q24h dose, considering a male individual with the average characteristics of the study population: 57 years old, 169 cm, CL = 6.07 L/h).

**Figure 6:**
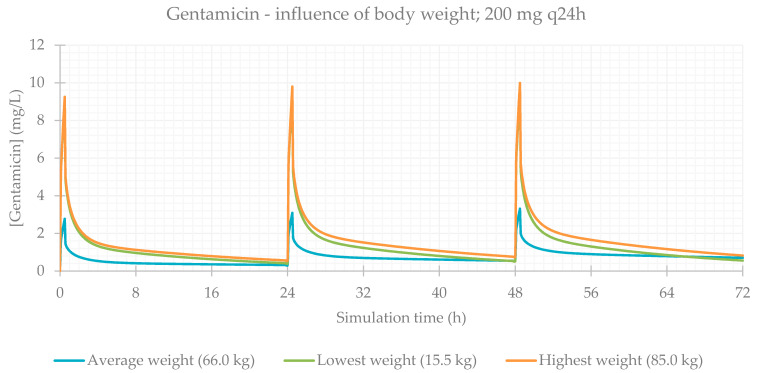
Gentamicin plasma concentration–time profile showing the influence of body weight (for a 200 mg q24h dose, considering a male individual with the average characteristics of the study population: 58 years old, 165 cm, CL = 5.94 L/h).

**Figure 7:**
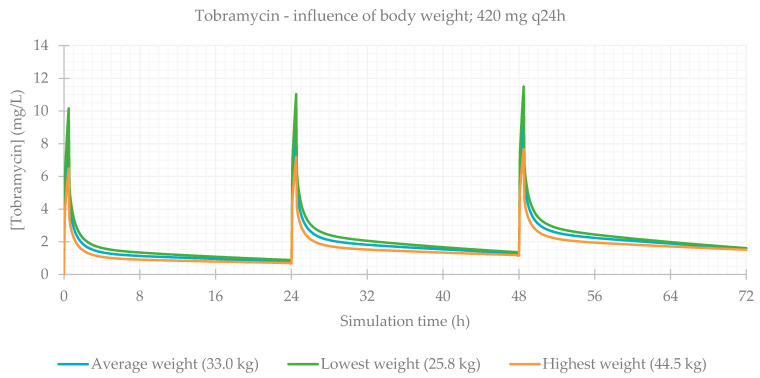
Tobramycin plasma concentration–time profile showing the influence of body weight (for a 420 mg q24h dose, considering a male individual with the average characteristics of the study population: 15 years old, 146 cm, CL = 6.01 L/h).

**Figure 8:**
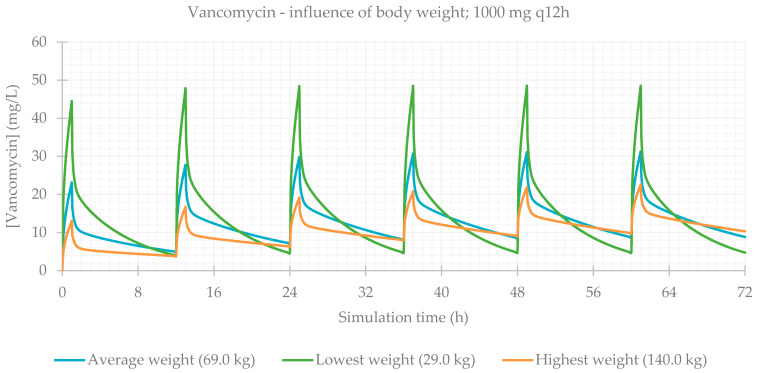
Vancomycin plasma concentration–time profile showing the influence of body weight (for a 1000 mg q12h dose, considering a male individual with the average characteristics of the study population: 63 years old, 163 cm, CL = 5.79 L/h).

Concerning body composition, the influence of total body weight was assessed, and the resulting PK parameters are presented in Table 4. As expected, simulations for patients with a lower weight result in a higher C_max_ and higher drug concentrations maintained throughout time. The plasma concentration–time profiles showing the influence of this parameter are shown for each antibiotic in Figure 5, Figure 6, Figure 7 and Figure 8.

### 3.3. Influence of Renal Function

**Figure 9:**
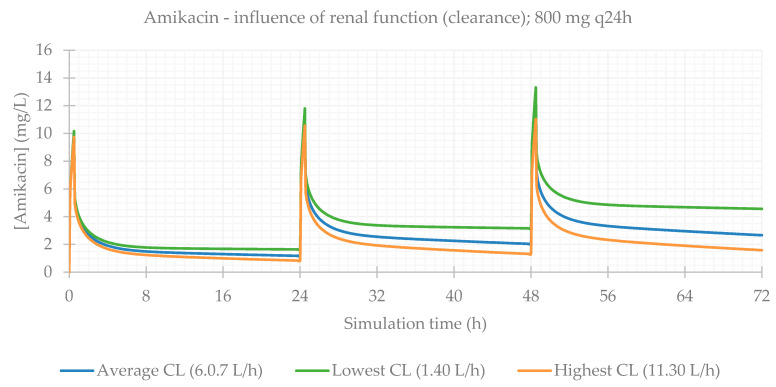
Amikacin plasma concentration–time profile showing the influence of renal function (for an 800 mg q24h dose, considering a male individual with the average characteristics of the study population: 57 years old, 66.0 kg, 169 cm).

**Figure 10:**
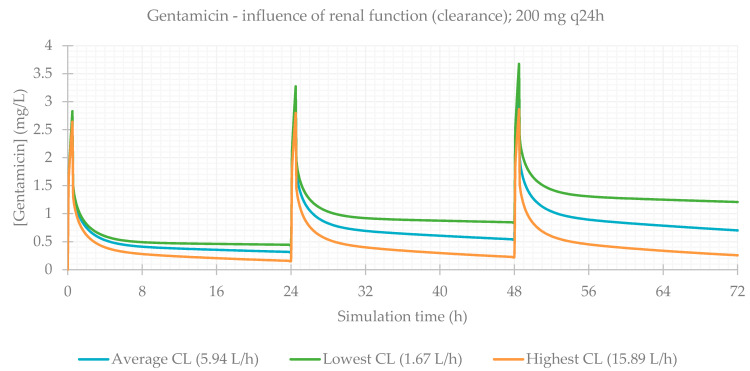
Gentamicin plasma concentration–time profile showing the influence of renal function (for a 200 mg q24h dose, considering a male individual with the average characteristics of the study population: 58 years old, 66.0 kg, 165 cm).

**Figure 11:**
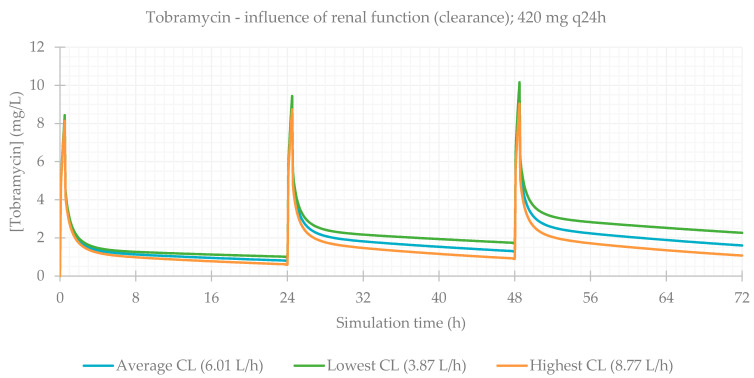
Tobramycin plasma concentration–time profile showing the influence of renal function (for a 420 mg q24h dose, considering a male individual with the average characteristics of the study population: 15 years old, 33.0 kg, 146 cm).

**Figure 12:**
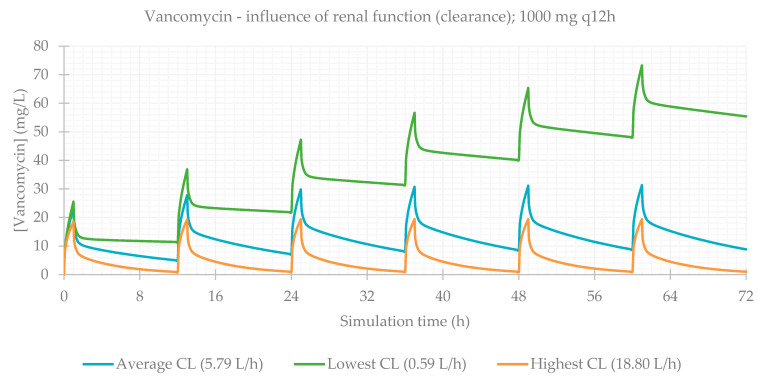
Vancomycin plasma concentration–time profile showing the influence of renal function (for a 1000 mg q12h dose, considering a male individual with the average characteristics of the study population: 63 years old, 69.0 kg, 163 cm).

Finally, the influence of renal function was evaluated. This is a particularly relevant consideration, since during treatment (especially for long periods of time), kidneys can be affected as a side effect of medication, and renal function is often impaired. The PBPK simulations confirmed this, and CL_Cr_ was the most significantly impacting factor on antibiotics plasma concentrations. PK parameters are presented in Table 5 and plasma concentration–time profiles are illustrated for each antibiotic in Figure 9, Figure 10, Figure 11 and Figure 12.

When considering the lowest CL_Cr_, antibiotic concentrations are much higher in comparison to individuals with normal functioning kidneys. This is exceptionally important in the case of vancomycin, where the differences between the lower and upper limits of observed CL_Cr_ were exceedingly significant.

### 3.4. Effect of Renal Function on the Accumulation of Drugs in Plasma over Time

Since the influence of renal function on the PK profile of the antibiotics was so significant, the observed differences were further analyzed.

**Figure 13:**
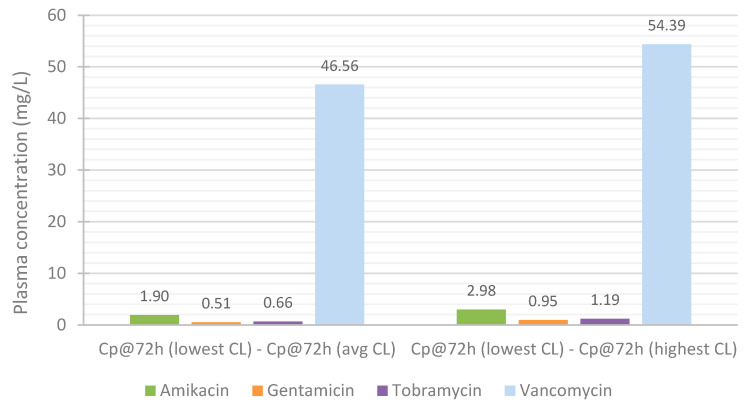
Influence of renal function on the accumulation of the antibiotics in plasma throughout treatment: difference in the plasma concentration at 72 h.

**Figure 14:**
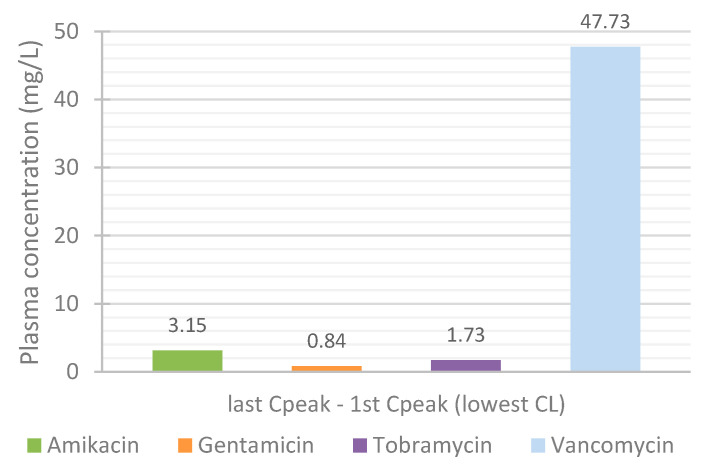
Influence of renal function on the accumulation of the antibiotics in plasma throughout treatment: difference between the C_peak_ of the last and C_peak_ of the first simulated administration.

The differences in the predicted antibiotics concentrations at 72 h between the simulation for the lowest and average CL, and lowest and highest CL were calculated (Figure 13). For the simulations customized to represent the patients with the lowest CL, the differences between the C_peak_ of the last and C_peak_ of the first administrations were also determined (Figure 14). As aforementioned, renal function has a major impact on the plasma concentration profile of antibiotic vancomycin. As such, it is particularly important to closely observe patients receiving this antibiotic, monitoring their drug’s plasma concentration and changes in their renal function. Vancomycin can easily accumulate and rapidly reach toxic levels in plasma, which can lead to severe adverse effects and cause permanent damage to the kidneys.

## 4. Conclusions

In this work, clinical data from inpatients with severe systemic infections were analyzed and PBPK simulations were performed to study the pharmacokinetic profile of four clinically used antibiotics: amikacin, gentamicin, tobramycin, and vancomycin. The importance of therapeutic drug monitoring and regimen adjustment was further demonstrated, by highlighting the influence of parameters such as a patient’s total body weight and the significant impact of renal function on antibiotic plasma concentrations. It was made evident that patients with low body weight and especially those with impaired renal function, whether preexisting or due to kidney deterioration throughout treatment, must be closely monitored and their therapeutic regimens frequently revised to adjust to their plasma antibiotic concentrations and PK profile. We also found that renal function is the parameter that has the greatest impact on the Cp-time profile of these antibiotics, especially vancomycin, but that there are certainly other factors affecting PK and more optimizations to be made to these PBPK models, since the values obtained in the simulations may come even closer to those observed clinically. In particular, it is known that hospitalized and critically ill patients have altered PK and the fact that in this project it was not possible, due to software limitation, to insert the observed concentrations into the models, refining, optimizing, and then validating them, also contributed to the slight differences between the simulated and clinical results.

The results presented here deepen the knowledge on the impact and influence of these parameters on the biodistribution of these antibiotics. This is crucial not only to ensure a successful clinical outcome, but also to prevent serious side effects, and, as such, can assist clinicians in the process of adjusting therapeutic regimens. In this context, the helpfulness of in silico tools, such as PBPK modeling and software such as GastroPlus™, was also verified.

## Figures and Tables

**Table 1:** Summary of collected clinical data, with the indication of the average value, lower and upper limits for each parameter. Normal range values (CHUP reference) are also presented.

	Amikacin	Gentamicin	Tobramycin	Vancomycin
n_t_ (n_sex_)	8 (F: 1; M: 7)	22 (F: 8; M: 14)	5 (F: 4; M: 1)	47 (F: 21; M: 26)
Age (years)	14–87 (avg 57)	7–88 (avg 58)	13–19 (avg 15)	19–93 (avg 63)
Weight (kg)	50.0–92.5 (avg 66.0)	15.5–85.0 (avg 66.0)	25.8–44.5 (avg 33.0)	29.0–140. (avg 69.0)
Height (cm)	163–180 (avg 169)	108–185 (avg 165)	130–158 (avg 146)	147–185 (avg 163)
C_min_ (mg/L)	0.30–16.40 (avg 3.70) (ref 1–8)	0.20–4.80 (avg 1.05) (ref 0.5–2)	0.06–0.23 (avg 0.17) (ref 0.5–2; <1 CF)	4.50–45.60 (avg 16.34) (ref 15–20)
C_max_ (mg/L)	19.70–87.80 (avg 38.97) (ref 20–30)	2.90–19.50 (avg 9.22) (ref 5–10)	16.32–36.12 (avg 27.05) (ref 5–10; 20–30 CF)	11.20–60.10 (avg 25.99) (ref < 40)
[Cr] (mg/dL)	0.47–1.58 (avg 0.93)	0.29–1.89 (avg 0.83)	0.35–0.64 (avg 0.49)	0.27–4.78 (avg 1.00)
CL (L/h) *	1.40–11.30 (avg 6.07)	1.67–15.89 (avg 5.94)	3.87–8.77 (avg 6.01)	0.59–18.80 (avg 5.79)

* Estimation using the Cockcroft–Gault equation. Ref: normal range (CHUP). CF: Cystic fibrosis.

**Table 2:** Physicochemical properties of antibiotics and input parameters in GastroPlus™.

	Amikacin	Gentamicin	Tobramycin	Vancomycin
Molecular weight	585.61	477.61	467.52	1449.28
logP	−5 ^1^	−1.79 ^1^; −3.1 ^2^	−4.8 ^1^; −5.8 ^2^	2.48 ^1^; −3.1 ^2^
pKa	8.1 ^2^	12.55; 10.18 ^2^	12.54; 9.83 ^2^	2.99; 9.93 ^2^
Solubility	166.49 g/L, pH = 11.73 ^1^; 50 g/L ^2^	56.54 g/L, pH = 11.51 ^1^; 12.6 g/L ^2^	59.47 g/L, pH = 11.34 ^1^; freely soluble ^2^	0.26 g/L, pH = 8.17 ^1^; 0.225 g/L ^2^
Diffusion Coefficient	0.53 cm^2^/s × 10^5 1^	0.56 cm^2^/s × 10^5 1^	0.6 cm^2^/s × 10^5 1^	0.32 cm^2^/s × 10^5 1^
Drug particle density	1.2 g/mL	1.2 g/mL	1.2 g/mL	1.2 g/mL
Mean particle radius	25	25	25	25
P_eff_	0.0202 cm/s × 10^−4 1^	0.0891 cm/s × 10^−4 1^	0.0368 cm/s × 10^−4 1^	0.0747 cm/s × 10^−4 1^
F_up_	98.75% ^1^; >90% ^2^	85.4% ^1^; >70% ^2^	100% ^1^; >70% ^2^	21.36% ^1^; ~50% ^2^
Blood/plasma ratio	1.23 ^1^	1.11 ^1^	1.31 ^1^	0.68 ^1^
T_1/2_	2–3 h	2–3 h	2–3 h	~6 h (4–11 h)
V_c_	0.24 ^1^; ~0.34 L/kg ^2^	0.38 ^1^; 0.2–0.3 L/kg	0.3 ^1^; 0.2–0.3 L/kg	0.4–1 L/kg
Clearance	6.00 L/h	3.42 L/h	8.48 L/h	4.03 L/h
Typical dosing for susceptible infections	7.5 mg/kg q12h 15 mg/kg q24h	1 mg/kg q8h	1 mg/kg q8h	1000 mg q12h
Common dosing in CHUP	Variable: 400–800 mg q24h or q48h	120 mg q8h 200 mg q24h 70 mg q12h	70 mg q8h 420 mg q24h	1 g q12h 1 g q24h

^1^ predicted by GastroPlus™; ^2^ from DrugBank and references [38,39,40,41,42,43,44].

**Table 3:** Influence of therapeutic regimens and biological sex on the pharmacokinetic parameters of the four studied antibiotics.

Drug & Posology	Fa (%) ^1^	FDp (%) ^2^	F (%) ^3^	C_max_ (mg/L) ^4^	T_max_ (h) ^5^	AUC_0-inf_ (µg∙h/mL) ^6^	AUC_0-t_ (µg∙h/mL) ^7^	C_max liver_ (mg/L) ^8^
Amikacin								
400 mg q24h	99.980	99.964	99.964	5.981	48.5	197.570	94.836	64.761
800 mg q24h	99.980	99.964	99.964	11.962	48.5	395.150	189.670	129.520
800 mg q24h female	99.978	99.960	99.960	11.790	48.5	395.060	209.520	128.610
Gentamicin								
70 mg q12h	99.859	99.834	99.834	1.446	60.5	68.971	32.731	14.831
120 mg q8h	99.864	99.838	99.838	2.981	64.5	163.850	81.287	31.975
200 mg q24h	99.845	99.822	99.822	3.304	48.5	100.650	51.345	31.876
200 mg q24h female	99.829	99.806	99.806	3.246	48.5	100.630	56.321	31.524
Tobramycin								
250 mg q24h	99.971	99.971	99.971	5.702	48.5	124.780	78.230	62.346
500 mg q24h	99.971	99.971	99.971	11.403	48.5	249.570	156.460	124.690
420 mg q24h	99.971	99.971	99.971	9.579	48.5	209.640	131.430	104.740
420 mg q24h female	99.969	99.969	99.969	9.460	48.5	209.640	139.840	104.770
Vancomycin								
500 mg q12h	99.929	99.921	99.921	15.632	61.0	517.990	452.870	30.871
1000 mg q24h	99.926	99.921	99.921	25.547	49.0	518.000	477.990	50.086
1000 mg q12h	99.929	99.921	99.921	31.263	61.0	1036.000	905.740	61.742
1000 mg q12h female	99.930	99.921	99.921	29.202	61.0	1036.000	901.460	57.773

^1^ fraction absorbed as a percent of the dose (crossing the lumen and entering enterocytes); ^2^ percent of the dose that has reached the portal vein; ^3^ bioavailability; ^4^ maximum plasma concentration reached in the central compartment, in mg/L; ^5^ time to reach maximum plasma concentration, in hours; ^6^ area under the plasma concentration–time curve, in µg∙h/mL, extrapolated to infinity; ^7^ area under the plasma concentration–time curve, in µg∙h/mL, for the time of the simulation; ^8^ maximum concentration reached in the liver, in mg/L.

**Table 4:** Influence of total body weight on the pharmacokinetic parameters of the four studied antibiotics.

Drug & Posology	Fa (%) ^1^	FDp (%) ^2^	F (%) ^3^	C_max_ (mg/L) ^4^	T_max_ (h) ^5^	AUC_0-inf_ (µg∙h/mL) ^6^	AUC_0-t_ (µg∙h/mL) ^7^	C_max liver_ (mg/L) ^8^
Amikacin								
800 mg q24h BW avg (66.0 kg)	99.980	99.964	99.964	11.962	48.5	395.150	189.670	129.520
800 mg q24h BW min (50.0 kg)	99.981	99.965	99.965	14.813	48.5	395.250	223.520	161.780
800 mg q24h BW max (92.5 kg)	99.982	99.966	99.966	9.161	48.5	394.940	151.390	97.834
Gentamicin								
200 mg q24h BW avg (66.0 kg)	99.845	99.822	99.822	3.304	48.5	100.650	51.345	31.876
200 mg q24h BW min (15.5 kg)	99.910	99.905	99.905	9.555	48.5	100.890	90.418	97.446
200 mg q24h BW max (85.0 kg)	99.680	99.656	99.656	9.546	48.5	100.630	90.200	97.126
Tobramycin								
420 mg q24h BW avg (33.0 kg)	99.971	99.971	99.971	9.579	48.5	209.640	131.430	104.740
420 mg q24h BW min (25.8 kg)	99.973	99.973	99.973	11.479	48.5	209.660	147.220	127.010
420 mg q24h BW max (44.5 kg)	99.970	99.970	99.970	7.646	48.5	209.600	111.740	82.333
Vancomycin								
1000 mg q12h BW avg (69.0 kg)	99.929	99.921	99.921	31.263	61.0	1036.000	905.740	61.742
1000 mg q12h BW min (29.0 kg)	99.956	99.954	99.954	48.459	61.0	1036.300	1005.000	95.749
1000 mg q12h BW max (140.0 kg)	99.936	99.926	99.926	22.535	61.0	1035.800	736.330	44.587

^1^ fraction absorbed as a percent of the dose (crossing the lumen and entering enterocytes); ^2^ percent of the dose that has reached the portal vein; ^3^ bioavailability; ^4^ maximum plasma concentration reached in the central compartment, in mg/L; ^5^ time to reach maximum plasma concentration, in hours; ^6^ area under the plasma concentration–time curve, in µg∙h/mL, extrapolated to infinity; ^7^ area under the plasma concentration–time curve, in µg∙h/mL, for the time of the simulation; ^8^ maximum concentration reached in the liver, in mg/L.

**Table 5:** Influence of renal function (creatinine clearance) on the pharmacokinetic parameters of the four studied antibiotics.

Drug & Posology	Fa (%) ^1^	FDp (%) ^2^	F (%) ^3^	C_max_ (mg/L) ^4^	T_max_ (h) ^5^	AUC_0-inf_ (µg∙h/mL) ^6^	AUC_0-t_ (µg∙h/mL) ^7^	C_max liver_ (mg/L) ^8^
Amikacin								
800 mg q24h CL avg (6.068 L/h)	99.980	99.964	99.964	11.962	48.5	395.150	189.670	129.520
800 mg q24h CL min (1.401 L/h)	99.974	99.950	99.950	13.293	48.5	1705.900	258.260	147.360
800 mg q24h CL max (11.298 L/h)	99.985	99.973	99.973	11.015	48.5	212.320	142.930	117.190
Gentamicin								
200 mg q24h CL avg (5.941 L/h)	99.845	99.822	99.822	3.304	48.5	100.650	51.345	31.876
200 mg q24h CL min (1.667 L/h)	99.789	99.751	99.751	3.667	48.5	355.600	69.999	36.212
200 mg q24h CL max (15.887 L/h)	99.908	99.899	99.899	2.860	48.5	37.744	30.152	26.809
Tobramycin								
420 mg q24h CL avg (6.007 L/h)	99.971	99.971	99.971	9.579	48.5	209.640	131.430	104.740
420 mg q24h CL min (3.868 L/h)	99.965	99.965	99.965	10.149	48.5	325.460	158.850	111.740
420 mg q24h CL max (8.771 L/h)	99.977	99.977	99.977	9.032	48.5	143.600	106.330	98.209
Vancomycin								
1000 mg q12h CL avg (5.787 L/h)	99.929	99.921	99.921	31.263	61.0	1036.000	905.740	61.742
1000 mg q12h CL min (0.590 L/h)	99.789	99.743	99.743	73.192	61.0	10090.000	2659.400	146.400
1000 mg q12h CL max (18.796 L/h)	99.976	99.974	99.974	19.356	61.0	319.140	313.790	38.136

^1^ fraction absorbed as a percent of the dose (crossing the lumen and entering enterocytes); ^2^ percent of the dose that has reached the portal vein; ^3^ bioavailability; ^4^ maximum plasma concentration reached in the central compartment, in mg/L; ^5^ time to reach maximum plasma concentration, in hours; ^6^ area under the plasma concentration–time curve, in µg∙h/mL, extrapolated to infinity; ^7^ area under the plasma concentration–time curve, in µg∙h/mL, for the time of the simulation; ^8^ maximum concentration reached in the liver, in mg/L.

## Data Availability

Not applicable.

## Supplementary Materials

The following are available online at https://www.mdpi.com/article/10.3390/life11111130/s1, Table S1: Demographic information and clinical data of the study population.
