# Supplementary material for: PBPK Modeling and Simulation of Antibiotics Amikacin, Gentamicin, Tobramycin, and Vancomycin Used in Hospital Practice
Source: Life (Basel). 2021 Oct 23;11(11):1130. doi: 10.3390/life11111130 (PMC8620954; doi:10.3390/life11111130)
Supplement: Supplementary file 1 [file life-11-01130-s001.zip › life-1387813-supplementary.pdf]

life

SUPPLEMENTARY MATERIALS

PBPK modeling and simulation of antibiotics amikacin, gentamicin, tobramycin, and vancomycin used in hospital practice

Abigail Ferreira [1,3], Helena Martins [2], José Carlos Oliveira [2], Rui Lapa [3] and Nuno Vale [1,4,*]

1 OncoPharma Research Group, Center for Health Technology and Services Research (CINTESIS), Rua Doutor Plácido da Costa, 4200-450 Porto, Portugal; abigail.ferreira@fc.up.pt
2 LAQV/REQUIMTE, Laboratory of Applied Chemistry, Department of Chemical Sciences, Faculty of Pharmacy, University of Porto, Rua de Jorge Viterbo Ferreira, 228, 4050-313 Porto, Portugal; laparuas@ff.up.pt
3 Departament of Pathology, Clinical Chemistry Service, Centro Hospitalar Universitário do Porto (CHUP), Largo Professor Abel Salazar, 4099-001 Porto, Portugal; helena.martins.sqc@chporto.min-saude.pt (H.M.); director.sqc@chporto.min-saude.pt (J.C.O.)
4 Department of Community Medicine, Health Information and Decision (MEDCIDS), Faculty of Medicine, University of Porto, Alameda Professor Hernâni Monteiro, 4200-319 Porto, Portugal
* Correspondence: nunovale@med.up.pt; Tel.: +351-220426537

Table S1. Demographic information and clinical data of the study population.
Blood samples were typically collected prior to the following antibiotic administration to determine C_{min} (at most 30 minutes before infusion) and 1 h (aminoglycosides) or 3 h (vancomycin) after the beginning of an infusion to determine C_{max}. When an occasional sample was collected, the measured concentration is presented with the indication of the time of blood sample collection. In some cases, it was not possible to determine the antibiotic concentration and a calculated range is presented (estimation of the software used in the clinical setting, PKS, ABBOTTBASE® Pharmacokinetic Systems, version 1.10, Abbott Laboratories, Texas, USA). The considered normal range of creatinine concentration in plasma is 0.7 - 1.2 mg/dL for male patients and 0.5 - 0.9 mg/dL for female patients. Clearance was calculated from creatinine concentration using the Cockcroft-Gault formula [1]: CL = ((140-age) x weight)/(72 x [creatinine]); multiply by 0.85 for female individuals; CL in mL/min, weight in kg, [creatinine] in mg/dL.

						Recommended posology					
#ID	Sex	Age (years)	Weight (kg)	Height (cm)	Date	Dose (mg)	Interval	C_{min} (mg/L)	C_{max} (mg/L)	[Cr] (mg/dL)	CL (L/h)
						AMIKACIN					
25	Male	67	72.5	163	Day 1	900	24 h				
					Day 3	750	24 h	2.1	60.2	0.66	6.91
					Day 6	750	24 h			0.63	7.24
					Day 7	800	24 h	2.7	33.3	0.72	6.34
26	Male	79	60	168	Day 1	450	12 h			1.10	2.77
					Day 4	450	12 h			1.00	3.05
					Day 5	Susp 1 dose > 450 mg q24h		16.4	44.0	1.11	2.75
					Day 6	450	24 h	5.7	34.3	0.99	3.08
					Day 11	450	24 h	4.0	36.2	1.09	2.80
28	Fem	87	50	167	Day 1					1.34	1.40
					Day 7	750	24 h			0.91	2.06
					Day 9	500	24 h	4.3	56.0	0.79	2.38
					Day 14	500	24 h	3.5	41.3	0.67	2.80
					Day 19	500	24 h	3.3	40.6	0.71	2.64
29	Male	14	53.8	168	Day 1	1500	24 h				
					Day 4	1100	24 h	0.5	87.8	0.50	11.30
					Day 8	1100	24 h	0.3	51.7	0.53	10.66
32	Male	77	70	170	Day 1	500	24 h			1.26	2.92
					Day 2	500	24 h			1.19	3.09
					Day 3	500	24 h			1.28	2.87
					Day 4	800	24 h	2.4	19.7	1.09	3.37
					Day 5	800	24 h			1.03	3.57
					Day 6	700	24 h	4.6	41.3	1.01	3.64
					Day 9	700	24 h			1.14	3.22
					Day 10	700	24 h			1.21	3.04
					Day 11	Susp 1 dose > 800 mg q48h		7.6	49.3	1.25	2.94
36	Male	18	53.6	168	Day 1	500	8 h				
					Day 5	Susp 3 doses > 850 mg q24h		2.5	calc 27-28	0.62	8.79
					Day 9	850	24 h	0.5	calc 43-45	0.62	8.79
					Day 13	850	24 h	0.6	47.2	0.58	9.40
37	Male	58	92.5	180	Day 1					1.54	4.44
					Day 3	500	24 h				
					Day 5	650	24 h		(occas 3.5 h) 13.4	1.22	5.60
					Day 8	650	24 h			1.06	6.45
					Day 9	800	24 h	2.7	24.5	0.95	7.19
					Day 10	800	24 h			1.02	6.70
					Day 12	800	24 h	3.1	(occas 2 h) 23.4	1.22	5.60
					Day 15	800	24 h	3.4	(occas 1.5 h) 39.1	1.11	6.16
					Day 17	800	24 h			1.22	5.60

					Recommended posology						
#ID	Sex	Age (years)	Weight (kg)	Height (cm)	Date	Dose (mg)	Interval	C_{min} (mg/L)	C_{max} (mg/L)	[Cr] (mg/dL)	CL (L/h)
					Day 18	800	24 h	4.2	39.2		
					Day 19	800	24 h			1.38	4.95
					Day 20	800	24 h			1.03	6.63
					Day 22	800	24 h			1.23	5.56
					Day 23	800	24 h	5.5	35.4	1.09	6.27
					Day 25	800	24 h			1.27	5.38
					Day 26	650	24 h	5.8	38.5	1.23	5.56
					Day 29	650	24 h			1.30	5.26
					Day 30	Susp 1 dose > 800 mg q48h		5.7	25.3	1.48	4.62
					Day 31					1.45	4.71
					Day 33	800	48 h			1.47	4.65
					Day 36	750	48 h	1.8	33.3	1.36	5.02
					Day 37	750	48 h			1.52	4.50
					Day 38	750	48 h			1.58	4.32
					Day 40	750	48 h	2.0	24.7	1.43	4.78
					Day 43	750	48 h			1.37	4.99
					Day 45	750	48 h			1.36	5.02
					Day 46	750	48 h	1.9	28.8		
38	Male	56	75	170	Day 1	800	24 h			0.74	7.09
					Day 2	800	24 h			0.82	6.40
					Day 3	800	24 h			0.75	7.00
					Day 4	800	24 h			0.58	9.05
					Day 5	800	24 h			0.55	9.55
					Day 6	800	24 h			0.54	9.72
					Day 7	800	24 h	4.7	34.4	0.61	8.61
					Day 8	800	24 h			0.51	10.29
					Day 9	800	24 h			0.63	8.33
					Day 10	800	24 h	calc 3-4	calc 31-33	0.60	8.75
					Day 13	400	12 h			0.50	10.50
					Day 18	650	24 h	4	23	0.50	10.50
					Day 19	650	24 h			0.47	11.17
					Day 20	550	24 h	5.6	(occas 1.5 h) 39.7	0.53	9.91
					Day 21	550	24 h			0.54	9.72
					Day 22	550	24 h			0.64	8.20
					Day 23	550	24 h			0.83	6.33
					Day 24	550	24 h			0.77	6.82
					Day 25	550	24 h			0.59	8.90
					Day 26	Susp 1 dose > 700 mg q48h		5.2	28.7	0.69	7.61
					Day 27	700	48 h			0.88	5.97
					Day 28	700	48 h			0.66	7.95
					Day 29	700	48 h			0.70	7.50
					Day 30	700	48 h			0.75	7.00
					Day 31	700	48 h			0.90	5.83
					Day 32	700	48 h	2.2	calc 31-33	0.86	6.10
					Day 33	700	48 h			0.92	5.71
					Day 34	700	48 h	2.0	34.5	0.76	6.91
					Day 35	700	48 h			0.70	7.50
					Day 36	700	48 h			0.71	7.39

					Recommended posology						
#ID	Sex	Age (years)	Weight (kg)	Height (cm)	Date	Dose (mg)	Interval	C_{min} (mg/L)	C_{max} (mg/L)	[Cr] (mg/dL)	CL (L/h)
					Day 37	700	48 h			0.59	8.90
					Day 38	700	48 h			0.74	7.09
					Day 39	700	48 h	1.3	(occas 1.3 h) 37.2	0.65	8.08
**GENTAMICIN**											
7	Male	49	67	175	Day 1					0.67	7.58
					Day 3	180	12 h			0.63	8.06
					Day 4	180	12 h	0.3	calc 9.5 - 10.5	0.63	8.06
					Day 6	220	12 h	0.3	6.7	0.52	9.77
					Day 10	220	12 h	0.6	(occas 1.5 h) 9.1	0.70	7.26
					Day 12	220	12 h	0.5	8.9	0.69	7.36
18	Fem	72	70	160	Day 1	210	24 h				
					Day 2	210	24 h			1.09	3.09
					Day 4	120	24 h	0.5	13.7		
					Day 5	120	24 h			1.08	3.12
					Day 7	120	24 h			1.16	2.91
					Day 8	100	24 h	0.4	7.1	1.30	2.59
19	Male	78	47	170	Day 1					0.61	3.98
					Day 2	240	24 h				
					Day 3	240	24 h			0.68	3.57
					Day 4	240	24 h				
					Day 5	240	24 h			0.65	3.74
					Day 6	350	24 h	0.8	(occas 1.5 h) 8.6	0.62	3.92
					Day 7	350	24 h	1.1	16.9	0.81	3.00
					Day 9	380	24 h	1.8	18.3	0.84	2.89
22	Fem	88	75	155	Day 1	300	24 h			0.68	4.06
					Day 5	Susp 2 doses > 160 mg q48h		4.2	19.17	0.81	3.41
					Day 8	160	48 h			0.90	3.07
					Day 12	Susp 3 doses > 170 mg q72h		1.7	9.9	1.42	1.95
23	Fem	60	65.3	165	Day 1	62	8 h		(occas 5 h) 1.7	0.48	7.59
					Day 2	150	12 h			0.64	5.69
					Day 3	150	12 h		(occas 5 h) 2.1	0.51	7.14
					Day 6	150	12 h			0.77	4.73
					Day 7	170	12 h	0.8	5.8	0.58	6.28
					Day 8	170	12 h			0.66	5.52
					Day 9	170	12 h			0.60	6.07
					Day 10	Susp 2 doses > 200 mg q24h		1.3	6.8 [a]	0.58	6.28
					Day 11	200	24 h				
					Day 13	200	24 h			0.71	5.13
					Day 14	150	24 h	0.8	10.6	0.70	5.21
					Day 16	150	24 h			0.82	4.44
					Day 20	Susp 1 dose > 120 mg q48h		1.3	9.9	1.23	2.96
					Day 22	120	48 h			0.90	4.05
					Day 24	150	48 h	0.2	5.1	0.73	4.99
					Day 26	100	24 h	0.1	5.1		
24	Fem	50	65	157	Day 1					0.96	4.32
					Day 4	100	8 h			0.84	4.93
					Day 8	Susp 1 dose > 130 mg q24h		3.8	10.0	0.91	4.55

					Recommended posology						
#ID	Sex	Age (years)	Weight (kg)	Height (cm)	Date	Dose (mg)	Interval	C_{min} (mg/L)	C_{max} (mg/L)	[Cr] (mg/dL)	CL (L/h)
					Day 9	130	24 h				
					Day 11	140	24 h	0.8	7.5	0.95	4.36
					Day 15	120	24 h	0.6	12.2	1.12	3.70
27	Male	20	70	175	Day 1	70	8 h			0.87	8.05
					Day 2	70	8 h			0.71	9.86
					Day 3	70	8 h			0.75	9.33
					Day 4	70	8 h			0.76	9.21
					Day 5	160	12 h	0.3	3.4	0.86	8.14
					Day 8	160	12 h	0.4	7.6	0.75	9.33
					Day 10	160	12 h	0.4	8.2	0.88	7.95
					Day 12	160	12 h	0.3	7.8	0.78	8.97
					Day 13	160	12 h			0.70	10.00
					Day 16	140	12 h	0.6	7.6	0.86	8.14
33	Male	75	53	170	Day 1					1.49	1.93
					Day 3	60	8 h			0.90	3.19
					Day 6	Susp 1 dose > 150 mg q24h		2.5	5.3	0.89	3.23
					Day 11	100	24 h	1.0	13.2		
					Day 13	100	24 h	0.6	8.2		
34	Male	69	83	180	Day 1					1.04	4.72
					Day 4	240	24 h			0.85	5.78
					Day 7	150	24 h	0.2	(occas 1.5 h) 9.1	0.97	5.06
					Day 11	150	24 h	0.2	4.6	1.04	4.72
					Day 12	170	24 h			1.10	4.46
					Day 14	160	24 h	0.2	(occas 1.5 h) 5.6	1.04	4.72
35	Male	50	80	185	Day 1	160	24 h				
					Day 6	190	12 h	0.2	6.6	0.49	12.24
					Day 8	Susp 1 dose > 200 mg q24h		1.1	10.4	0.51	11.76
					Day 15	200	24 h	0.4	9.4	0.52	11.54
					Day 21	Susp 1 dose > 190 mg q24h		1.9	12.8	0.65	9.23
					Day 23	190	24 h	0.5	10.8	0.82	7.32
					Day 27	190	24 h			1.14	5.26
					Day 28	Susp 2 doses > 180 mg q48h		2.2	13.4	1.47	4.08
50	Male	73	80	170	Day 1	200	24 h				
					Day 7	150	24 h	1.2	calc 10-12	1.14	3.92
62	Fem	7	15.5	108	Day 1	80	24 h			0.68	2.15
					Day 3	80	24 h	(occas) 0.4	calc 10-11	0.37	3.95
63	Male	62	85	180	Day 1	433.5	24 h				
					Day 8	433.5	24 h			0.76	7.27
					Day 9	433.5	24 h			0.72	7.67
					Day 10	500	24 h	0.3	19.5	0.73	7.57
-1	Male	86	70	165	Day 1	70	12 h	1.2	4.1	1.89	1.67
-2	Male	33	70	170	Day 1	80	8 h	0.3	3.1	0.59	10.58
-3	Male	60	85	161	Day 1	140	8 h	2.9	9.0	0.85	6.67
-4	Fem	78	44	160	Day 1	130	24 h	0.6	5.2	0.74	2.61
-5	Male	55	65	165	Day 1	120	8 h	0.2	2.9	0.29	15.88
-6	Male	34	71.4	165	Day 1	240	24 h	0.5	5.9	0.61	10.34
-7	Fem	73	62	165	Day 1	180	24 h	1.3	11.1	1.00	2.94

						Recommended posology					
**#ID**	**Sex**	**Age (years)**	**Weight (kg)**	**Height (cm)**	**Date**	**Dose (mg)**	**Interval**	**C_{min} (mg/L)**	**C_{max} (mg/L)**	**[Cr] (mg/dL)**	**CL (L/h)**
-8	Male	56	72	170	Day 1	210	24 h	4.8	14.4	1.00	5.04
-9	Fem	51	63	160	Day 1	180	24 h	0.2	6.6	0.64	6.21
						**TOBRAMYCIN**					
					Day 1	400	24 h				
					Day 3	400	24 h	0.2	18.8	0.51	7.20
30	Fem	13	40.8	146	Day 5	500	24 h				
					Day 7	450	24 h	---	33.7	0.49	7.49
					Day 12	450	24 h	0.23	calc 24-26	0.46	7.98
					Day 1	270	24 h				
31	Fem	13	27	140	Day 3	270	24 h	0.2	25.9	0.49	4.96
					Day 7	270	24 h	---	26.4	0.41	5.92
					Day 1	250	24 h				
39	Fem	13	25.8	130	Day 6	250	24 h	0.2	29.4	0.48	4.84
					Day 9	250	24 h	0.1	24.1	0.60	3.87
					Day 13	250	24 h	0.1	---	0.55	4.22
					Day 1	300	24 h			0.40	6.21
					Day 2	300	24 h			0.43	5.78
					Day 3	300	24 h	(occas) 0.2	calc 29-30	0.43	5.78
					Day 6	300	24 h	(occas) 0.1	calc 28-30		
40	Fem	19	29	155	Day 8	300	24 h	(occas) 0.1	calc 28-30	0.35	7.10
					Day 12	350	24 h	---	17.0	0.64	3.88
					Day 16	350	24 h	---	16.3	0.47	5.29
					Day 19	300	24 h	---	34.8	0.39	6.37
					Day 23	300	24 h	---	36.1	0.54	4.60
					Day 1	430	24 h				
41	Male	17	44.5	158	Day 7	400	24 h	0.2	35	0.52	8.77
					Day 10	400	24 h	0.2	calc 29-30	0.58	7.86
						**VANCOMYCIN**					
					Day 1					0.95	2.56
					Day 4	1000	12 h			0.90	2.70
1	Fem	88	66	155	Day 6	750	12 h	17.7	31.0	0.80	3.04
					Day 8	Susp 1 dose > 500 mg q12h		23.4	34.1	0.81	3.00
					Day 12	Susp 1 dose > 750 mg q24h		22.1	27.3	1.04	2.34
					Day 20	500	24 h	21.2	34.7	1.18	2.06
					Day 1	1000	12 h				
					Day 3	800	8 h	9.3	19.8	0.86	6.60
					Day 4					0.72	7.88
2	Fem	51	90	165	Day 6	800	8 h	10.2	19.1	0.55	10.32
					Day 9					0.74	7.67
					Day 10					0.79	7.18
					Day 11	950	8 h	12.2	21.2	0.60	9.46
					Day 1	1000	12 h				
					Day 8	Susp 1 dose > 600 mg q12h		26.0	36.1	1.05	5.46
3	Fem	50	80	165	Day 11	600	12 h	15.6	24.3	1.12	5.12
					Day 16	700	12 h	12.5	23.9	0.90	6.38
					Day 19	600	12 h	18.6	(occas 4 h) 29.7	1.02	5.63
4	Fem	61	71.5	160	Day 1					0.72	5.28

					Recommended posology						
**#ID**	**Sex**	**Age (years)**	**Weight (kg)**	**Height (cm)**	**Date**	**Dose (mg)**	**Interval**	**C_{min} (mg/L)**	**C_{max} (mg/L)**	**[Cr] (mg/dL)**	**CL (L/h)**
					Day 2	1000	12 h			0.63	6.04
					Day 3	1000	8 h	6.6	16.1	0.52	7.32
					Day 4					0.58	6.56
					Day 5					0.49	7.77
					Day 6	Susp 1 dose > 700 mg q8h		23.8	36.2	0.45	8.46
					Day 9	700	8 h	7.8	18.9	0.56	6.79
5	Male	58	87	185	Day 1	1000	12 h				
					Day 3					0.71	8.37
					Day 4	1000	8 h	7.3	16.8	0.72	8.26
					Day 6	Suspend administration		16.7	23.7	1.32	4.50
6	Male	62	71.9	172	Day 1	1000	12 h				
					Day 2					0.57	7.98
					Day 3	650	6 h	8.5	17.1	0.52	8.75
					Day 5	700	6 h	10.8	15.2	0.53	8.58
8	Male	53	65	165	Day 1					0.97	4.86
					Day 2	1000	12 h			1.16	4.06
					Day 5	850	12 h	calc 19-21	(occas 4 h) 30.2	0.88	5.36
					Day 6	850	12 h	15.4	27.3	1.10	4.28
					Day 7	850	12 h	14.9	28.4	1.01	4.67
					Day 9					1.23	3.83
					Day 13	600	12 h	22.3	34.0	1.13	4.17
9	Fem	66	140	163	Day 1	1000	12 h				
					Day 3	Susp 1 dose > 500 mg q12h		19.6	28.3		
					Day 7	Susp 1 dose > 950 mg q24h		17.4	22.9	0.84	8.74
					Day 13	800	24 h	14.1	(occas 4 h) 23.7		
10	Male	64	70	168	Day 1	1000	12 h				
					Day 5	1000	12 h	14.7	28.0		
					Day 7	850	12 h	19.3	21.1	1.17	3.79
					Day 12	Susp 1 dose > 1000 mg q24h		28.0	33.4	1.29	3.44
					Day 15	500	12 h	10.7	calc 27-29	1.35	3.28
11	Fem	47	65	147	Day 1	1000	12 h				
					Day 3	Susp 1 dose > 750 mg q12h		17.3	42.5	1.10	3.89
					Day 7	500	8 h	10.5	23.0		
					Day 10	500	8 h	13.1	22.4	0.70	6.12
12	Fem	73	80	160	Day 1	1000	12 h				
					Day 6	Susp 1 dose > 650 mg q12h		26.4	32.6	0.53	7.16
					Day 14	600	12 h	19.1	25.3		
					Day 19	600	12 h	15.5	23.7		
					Day 23	650	12 h	16.5	---		
					Day 25					0.47	8.08
					Day 27	600	12 h	20.9	26.6	0.45	8.44
					Day 30	500	12 h	22.4	31.0	0.41	9.26
14	Male	59	85	174	Day 1	500	6 h				
					Day 3	700	6 h	10.7	11.8		
					Day 4					0.48	11.95
					Day 5	800	6 h	11.4	14.8		
15	Fem	20	29	155	Day 1	1000	12 h				

						Recommended posology					
**#ID**	**Sex**	**Age (years)**	**Weight (kg)**	**Height (cm)**	**Date**	**Dose (mg)**	**Interval**	**C_{min} (mg/L)**	**C_{max} (mg/L)**	**[Cr] (mg/dL)**	**CL (L/h)**
					Day 3	500	6 h	5.8	---	0.35	7.04
					Day 5	300	4 h	8.1	15.0	0.35	7.04
					Day 8	300	4 h	13.2	---	0.41	6.01
					Day 11	300	4 h	1.6[a]; calc 12-14		0.40	6.16
					Day 1					4.78	0.63
					Day 2	900	12 h				
					Day 3					4.46	0.67
					Day 4					4.43	0.68
					Day 5	Suspend administration		22.3	(occas 4 h) 39.6	3.99	0.75
16	Male	80	60	160	Day 6					2.98	1.01
					Day 7			24.3	---	3.32	0.90
					Day 8	500	24 h			2.83	1.06
					Day 9					2.24	1.34
					Day 11					1.78	1.69
					Day 12	400	24 h	21.4	27.5	1.95	1.54
					Day 1					0.93	3.09
					Day 2					0.80	3.59
					Day 3					0.89	3.23
					Day 4					0.78	3.69
17	Fem	82	70	165	Day 5	1000	12 h				
					Day 6	1000	12 h	10.6	23.0	0.80	3.59
					Day 9					1.01	2.85
					Day 11	900	12 h	17.2	32.5	0.96	3.00
					Day 13	Susp 1 dose > 500 mg q12h		27.2	36.5	0.92	3.13
					Day 1	1000	12 h			0.50	10.15
					Day 3					0.40	12.69
					Day 4	850	6 h	4.8	11.2	0.40	12.69
21	Male	53	70	170	Day 6	850	6 h	9.6	14.2	0.54	9.40
					Day 7	900	6 h	9.5	13.0	0.40	12.69
					Day 9					0.68	7.46
					Day 10					0.78	6.51
					Day 11	850	6 h	18.5	28.1	0.68	7.46
					Day 1					3.32	0.59
					Day 2					2.85	0.69
42	Male	93	50	170	Day 4	1000	12 h				
					Day 8	Susp 3 days > 900 mg q24h		44.5	60.1	2.04	0.96
					Day 13	Suspend administration		31.4	45.3	2.06	0.95
					Day 1	1000	12 h				
					Day 6	750	6 h	7.9	15.6	0.61	6.66
43	Male	65	65	170	Day 9	850	6 h	12.9	17.7	0.69	5.89
					Day 13	Susp 2 doses > 650 mg q6h		21.1	27.9	0.74	5.49
					Day 22	1000	12 h				
					Day 26	600	6 h	11.0	21.3		
					Day 1	1000	8 h				
44	Male	54	70	172	Day 2	850	6 h	10.7	19.8		
					Day 4	800	6 h	13.7	21.9		

						Recommended posology					
**#ID**	**Sex**	**Age (years)**	**Weight (kg)**	**Height (cm)**	**Date**	**Dose (mg)**	**Interval**	**C_{min} (mg/L)**	**C_{max} (mg/L)**	**[Cr] (mg/dL)**	**CL (L/h)**
					Day 6					0.47	10.67
					Day 7					0.41	12.24
					Day 8	800	8 h	5.4	---	0.49	10.24
					Day 9					0.61	8.22
					Day 10	700	8 h	21.5[a]	---	0.53	9.47
					Day 11			9.5	14.2		
					Day 12					0.47	10.67
					Day 14					0.37	13.56
					Day 15			14.7		0.45	11.15
45	Male	61	90	175	Day 1	1000	12 h				
					Day 3					1.01	5.87
					Day 4					0.93	6.37
					Day 7	Suspend administration		14.9	30.2	0.81	7.31
46	Fem	70	61	158	Day 1	1000	12 h			0.77	3.93
					Day 3					0.74	4.09
					Day 6	700	12 h	20.5	35.6	0.76	3.98
47	Male	79	60	150	Day 1					1.98	1.54
					Day 3					1.98	1.54
					Day 4	1000	24 h				
					Day 8	Suspend administration		25.7	41.5	2.08	1.47
48	Fem	90	45	160	Day 1					1.14	1.40
					Day 4	700	12 h			1.19	1.34
					Day 7	500	12 h	21.0	30.3	1.11	1.44
					Day 10	500	12 h	---	29.5	0.97	1.64
					Day 16	Susp 2 doses > 800 mg q24h		20.3	---	1.19	1.34
					Day 18					1.38	1.15
					Day 21	500	24 h	22.1	---	1.49	1.07
49	Fem	85	80	163	Day 1	500	12 h				
					Day 2					1.20	2.60
					Day 3	Susp 2 doses > 650 mg q24h		25.4	---	0.83	3.76
					Day 4	650	24 h	16.8	25.8		
51	Fem	63	70	165	Day 1					0.54	7.07
					Day 2	1000	12 h				
					Day 3					0.93	4.11
					Day 4	Susp 1 dose > 650 mg q8h		12.3	16.3	0.86	4.44
52	Male	71	60	165	Day 1					1.36	2.54
					Day 3	1000	12 h			1.04	3.32
					Day 5	Susp 1 dose > 600 mg q12h		23.3	36.0	0.89	3.88
53	Male	59	70	180	Day 1	1000	12 h				
					Day 4	Susp 1 dose > 500 mg q12h		32.2	44.5		
					Day 5					1.22	3.87
					Day 8			17.9	22.4	1.17	4.04
					Day 9	850	6 h				
					Day 15	1000	12 h				
					Day 18					1.11	4.26
					Day 20					1.06	4.46
					Day 22	Suspend administration		45.6	57.1	1.25	3.78

Recommended posology											
#ID	Sex	Age (years)	Weight (kg)	Height (cm)	Date	Dose (mg)	Interval	C_{min} (mg/L)	C_{max} (mg/L)	[Cr] (mg/dL)	CL (L/h)
					Day 25			27.2	---		
54	Male	23	60	163	Day 1	1000	12 h				
					Day 3	850	6 h	5.1	---	0.88	6.65
					Day 6	850	6 h	12.0	---	1.02	5.74
					Day 10					1.00	5.85
					Day 13	900	6 h	10.2	---	0.96	6.09
					Day 17	900	6 h	17.0	---	0.98	5.97
					Day 20	900	6 h	9.6 [a]	---	0.94	6.22
55	Male	78	70	172	Day 1	1000	12 h				
					Day 7			21.0	28.0	0.83	4.36
					Day 8	750	12 h			0.94	3.85
					Day 9			22.1	---	0.82	4.41
					Day 10					0.86	4.21
					Day 11					0.82	4.41
					Day 12			16.9	---	0.93	3.89
					Day 15			16.9	25.9		
					Day 27	1000	12 h				
					Day 34			25.0	34.7		
					Day 35			18.5	29.4		
					Day 40	Suspend administration		26.2	37.6		
					Day 42				(occas) 11.4	0.86	4.21
56	Male	53	70	180	Day 1					0.35	14.50
					Day 5	1000	12 h			0.27	18.80
					Day 11			5.3	12.8	0.38	13.36
					Day 14	850	8 h	6.7	15.0	0.30	16.92
					Day 18	950	8 h	14.8	15.4		
					Day 20	800	6 h	12.4	19.8	0.32	15.86
57	Male	41	75	175	Day 1	1000	12 h				
					Day 3	900	12 h	16.7	25.2	1.55	3.99
					Day 8	Susp 2 doses > 600 mg q12h		24.9	33.8	1.44	4.30
					Day 11	550	12 h	17.8	23.1	1.68	3.68
					Day 14	600	12 h	16.1	21.9	1.20	5.16
					Day 18	650	12 h	14.4	28.6	1.01	6.13
					Day 22	650	12 h	16.7	26.4	1.02	6.07
					Day 25	650	12 h	16.0	44.3 [a]	1.02	6.07
58	Male	81	95	170	Day 1	1000	12 h				
					Day 2	800	12 h	8.4	---	1.34	3.49
					Day 4	600	12 h	25.7	34.0		
					Day 5	Susp 3 doses > 1000 mg q24h		27.1	31.7	1.39	3.36
					Day 6					1.25	3.74
					Day 8	1000	24 h	16.3	---	1.19	3.93
					Day 11			19.0	---		
					Day 12					1.11	4.21
					Day 15			23.3	42.6	1.17	3.99
					Day 16	Suspend administration		25.8		1.28	3.65
59	Fem	52	68	164	Day 1	1000	12 h				
					Day 6	700	8 h	12.7	---	0.70	6.06

					**Recommended posology**						
**#ID**	**Sex**	**Age (years)**	**Weight (kg)**	**Height (cm)**	**Date**	**Dose (mg)**	**Interval**	**C_{min} (mg/L)**	**C_{max} (mg/L)**	**[Cr] (mg/dL)**	**CL (L/h)**
60	Male	54	70	165	Day 1	1000	12 h				
					Day 6			17.6	---		
					Day 7					0.96	5.23
					Day 8					1.08	4.65
					Day 9			23.2	---	0.89	5.64
					Day 10	600	12 h	18.0	---	0.82	6.12
					Day 17	Susp 1 dose > 750 mg q24h		calc 24-26	32.5		
					Day 18					1.25	4.01
					Day 19					0.97	5.17
					Day 20	Susp 1 dose > 1000 mg q24h[b]		13.3	23.2	0.93	5.39
					Day 23	450	12 h	11.9	---		
					Day 24					0.63	7.96
					Day 25					0.64	7.84
					Day 26					0.65	7.72
					Day 27					0.56	8.96
61	Male	62	121	176	Day 1	1000	12 h				
					Day 4	1000	8 h	6.5	14.9	0.76	10.35
					Day 7	Susp 1 dose > 800 mg q6h		12.0	20.0	0.82	9.59
					Day 11					0.87	9.04
					Day 12	1000	12 h	16.0	19.7		
					Day 19	750	6 h	8.1	14.5	0.77	10.21
					Day 21	800	6 h	12.3	14.4[a]	0.79	9.96
					Day 25	900	6 h	12.2	15.1	0.73	10.77
					Day 29			14.9	20.1	0.82	9.59
					Day 31					0.87	9.04
					Day 33	900	6 h	16.9	20.5	0.77	10.21
					Day 36	Susp 2 doses > 800 mg q6h		11.6	16.9	0.84	9.36
					Day 39	900	6 h	17.4	21.2	0.80	9.83
64	Male	62	65	170	Day 1	500	6 h				
					Day 10					1.38	3.06
					Day 15	600	6 h	13.4	17.8	0.94	4.49
65	Fem	80	60	165	Day 1	1000	12 h			0.62	4.11
					Day 3	600	6 h	7.2	17.4	0.40	6.38
					Day 6	600	6 h	13.2	16.7	0.34	7.50
					Day 8	500	6 h	16.8	20.8	0.32	7.97
66	Fem	86	50	155	Day 1	1000	12 h	19.2	35.5	0.65	2.94
67	Fem	20	50	157	Day 1	1000	12 h	4.5	14.9	0.43	9.88
68	Male	54	58	160	Day 1	1000	12 h	15.0	34.6	0.63	6.60
69	Fem	46	70	156	Day 1	1000	12 h	6.6	19.2	0.38	12.27
70	Fem	78	47	160	Day 1	650	12 h	14.9	29.0	0.74	2.79
71	Fem	63	70	170	Day 1	1000	12 h	9.0	19.2	0.55	6.94
72	Male	83	70	170	Day 1	1000	12 h	15.1	26.9	0.97	3.43
73	Male	75	57	170	Day 1	1000	12 h	24.9	41.0	1.03	3.00
74	Male	83	65	170	Day 1	1000	12 h	22.3	36.8	1.28	2.41
75	Fem	19	60	170	Day 1	1000	8 h	18.0	43.1	1.87	2.75

[a] probable irregularity in the analytical determination of antibiotic concentration.
[b] previous posology was followed.

Susp X dose: X number of scheduled doses were suspended and recommended posology for when antibiotic administrations were resumed is presented.

Reference

Cockcroft, D.W.; Gault, M.H. Prediction of creatinine clearance from serum creatinine. *Nephron* **1976**, *16*, 31–41.
